# Machine Learning-Driven Paradigm for Polymer Aging Lifetime Prediction: Integrating Multi-Mechanism Coupling and Cross-Scale Modeling

**DOI:** 10.3390/polym17222991

**Published:** 2025-11-11

**Authors:** Bing Zeng, Shuo Wu, Shufang Yao

**Affiliations:** CABR Testing Center Company Limited, China Academy of Building Research, Beijing 100013, China

**Keywords:** aging life prediction, polymer, support vector machine, neural network model, decision tree model

## Abstract

This review systematically examined the transformative role of machine learning in predicting polymer aging lifetime, addressing critical limitations of conventional methods such as the Arrhenius model, time–temperature superposition principle, and numerical fitting approaches. The primary objective was to establish a comprehensive framework that integrates multi-mechanism coupling with dynamic data-driven modeling to enhance prediction accuracy across complex aging scenarios. Four key machine learning categories demonstrate distinct advantages: support vector machines effectively capture nonlinear interactions in multi-stress environments; neural networks enable cross-scale modeling from molecular dynamics to macroscopic failure; decision tree models provide interpretable feature importance quantification; and hybrid approaches synergistically combine complementary strengths. These methodologies have shown significant success in critical industrial applications, including building trades, photovoltaic systems, and aerospace composites, creating an integrated predictive system that bridges molecular-level dynamics with service-life performance. By transforming life prediction from empirical extrapolation to mechanism-based simulation, this machine-learning-driven paradigm offers robust methodological support for engineering safety design in diverse polymer applications through its capacity to model complex environmental interactions, adapt to real-time monitoring data, and elucidate underlying degradation mechanisms.

## 1. Introduction

Nowadays, polymer materials play a pivotal role in the advancement of modern civilization and are increasingly supplanting conventional materials across various fields, including medicine [1], construction [2], automotive [3,4], food [5], chemical [6] and mechanical manufacturing industries [7]. Over the past few decades, polymer materials have significantly propelled economic development by their superior durability, facile processing capabilities, diverse functionality and low production costs, thereby conferring immense benefits to our lives [8].

The exceptional mechanical, physical, and chemical properties of polymer materials could be preserved for extended periods to ensure their long-term stability in industrial applications. However, over time, polymer materials might undergo aging phenomena such as excessive hardening [9] or softening [10], cracking [11], or other surface damages [12] due to internal thermodynamic equilibrium changes and external factors like temperature variations, humidity levels, corrosive environments, exposure to ultraviolet light, and stress. Failure to address these issues could lead to potential risks. Therefore, during the design, development, and industrial application process of polymer materials, the investigation into predicting the aging life becomes particularly crucial [13].

The accurate prediction of the service life of polymer materials is crucial in terms of safety, particularly for key components and polymer products used in hazardous environments such as nuclear reactions and military applications, as it can effectively prevent catastrophic damage [14,15,16]. Moreover, life prediction plays a critical role in the research, development, and utilization of polymer materials across various fields, including medicine, engineering, and consumer goods production [17].

Although traditional methods such as the Arrhenius method, the time–temperature superposition principle (TTSP), and numerical fitting methods have played significant roles in predicting the lifespan of polymers, their inherent limitations increasingly restrict their applicability in complex service scenarios. The Arrhenius method considers temperature as the sole variable, neglecting the nonlinear modulation of activation energy by multi-mechanism coupling (such as the synergistic effect of wet heat and the interaction between light and stress). The “Thermorheological Simplicity” assumption on which TTSP relies fails in phase transition regions (such as near Tg) due to sudden changes in molecular relaxation patterns. Numerical fitting methods rely on manual parameter adjustment and cannot integrate real-time monitoring data for updated predictions. All three methods struggle to characterize the environment–material interaction effects (such as the synergistic chain scission and hydrolysis triggered by ultraviolet irradiation), leading to a sharp decline in confidence when making long-term extrapolations. These common bottlenecks highlight the insufficiency of traditional frameworks in capturing the complexity of polymer aging, urgently calling for the development of a new paradigm through machine learning that integrates multi-mechanism coupling modeling and dynamic data-driven approaches.

Consequently, machine learning methods (including artificial neural networks, random forests, and support vector machines) are increasingly employed to address aging scenarios involving ultraviolet exposure, humid heat, microbial degradation, and stress-induced deterioration. These data-driven approaches demonstrate superior capability in modeling multi-factor synergistic effects, overcoming the limitations of temperature-centric assumptions inherent in conventional models. To systematically frame these complexities, we classify the primary aging mechanisms into four categories: (1) thermo-oxidative aging, driven by temperature and oxygen interactions; (2) hydrolytic aging, induced by moisture and chemical media; (3) photo-aging, resulting from ultraviolet or light exposure; and (4) mechanical aging, involving stress, fatigue, and creep. Corresponding material defects include macroscopic failures such as cracking, hardening/softening, and discoloration, as well as microscopic defects like chain scission, crosslinking, and pore formation. Key influencing factors encompass environmental parameters (e.g., temperature, humidity, UV dose), material properties (e.g., molecular weight, functional groups), and mechanical stresses (e.g., cyclic loading, impact). Machine learning algorithms, particularly through their ability to fuse heterogeneous data sources (e.g., material properties, environmental parameters, and real-time monitoring signals), enable nonlinear mapping between these degradation mechanisms and service life. However, challenges persist regarding model interpretability, generalization across material formulations, and dependency on high-quality training datasets.

This review systematically analyzed the latest progress in polymer lifetime prediction methods, evaluated their applicability through comparative case studies, and proposed an innovative framework that integrates physical constraints with data-driven intelligence. The work is motivated by three fundamental scientific gaps in the current research landscape: (1) the inability of traditional models to capture multi-mechanism coupling effects under complex service conditions; (2) the lack of cross-scale modeling frameworks connecting molecular dynamics to macroscopic failure; and (3) the absence of a standardized methodology for selecting context-appropriate machine learning models. To address these gaps, this review establishes three specific aims: first, to critically evaluate the capabilities and limitations of major machine learning approaches (support vector machine, neural networks, decision trees, and hybrid models) in handling polymer aging complexity; second, to explore the feasibility and improvement of integrating multiple machine learning methods; and third, to propose a forward-looking roadmap guiding the development of standardized datasets, algorithm integration, and industrial validation for the physics–machine learning hybrid paradigm. By establishing this methodological foundation, the review aims to provide researchers with both a comprehensive technical reference and a clear development path for achieving reliable life cycle management of polymer materials.

The overall structure of this paper is centered around the above-mentioned goal. Chapter Two reviews traditional methods and their limitations. Chapter Three elaborates on the new paradigm of machine learning and critically analyzes its advantages and disadvantages. Chapter Four summarizes and looks forward, aiming to provide researchers with a comprehensive and profound academic reference.

## 2. Challenges in Conventional Lifespan Prediction of Polymeric Materials: Mechanisms and Shortcomings

In the field of polymer life prediction, traditional methods such as the Arrhenius model, TTSP, and numerical simulation have long dominated scientific research and engineering applications, but they are increasingly showing insufficient adaptability to complex aging scenarios.

### 2.1. Arrhenius Model

In the long-term durability assessment system of polymers, the traditional temperature-accelerated aging model based on the Arrhenius equation has long held a dominant position [13,18,19]. The core principle of this method relies on chemical reaction kinetics. The service life of materials under usage conditions can be predicted by establishing the exponential dependence of the reaction rate constant on temperature under high-temperature accelerated aging conditions, i.e., linear regression of the Arrhenius plot, and extrapolating this relationship to the target usage temperature [20].

However, the core limitation of this approach lies in its highly simplified model framework, which isolates temperature as the sole dominant variable driving polymer degradation. In actual service environments, polymers undergo complex processes involving the synergistic coupling of multiple physical fields such as heat, oxygen, moisture, light, and stress. For instance, thermal oxidation reactions and hydrolysis reactions often intertwine and mutually promote each other. Crucially, when polymers pass through their glass transition temperature (Tg) region, their molecular chain segment mobility undergoes a significant leap, causing the activation energy for aging reactions to no longer be a single value (i.e., not a single slope on an Arrhenius plot), but rather to undergo a sudden change. This discontinuity in activation energy makes the extrapolation of a single activation energy from the high-temperature region (T > Tg) to the service temperature region (T < Tg) inevitably lead to systematic and often significant deviations in predictions. Therefore, the Arrhenius method is inherently difficult to accurately describe the aging behavior of polymers under real service conditions, especially when phase transitions and multi-factor synergistic effects are involved.

### 2.2. Time–Temperature Superposition Principle

TTSP offers another classic approach to addressing the time-scale issue required for predicting the long-term performance of polymers. Its methodology aims to construct a so-called “master curve” that spans a wide range of time scales by horizontally shifting the short-term (such as mechanical relaxation and creep) curves measured at different temperatures, thereby inferring the ultra-long-term performance evolution of the material at low temperatures [21,22].

However, the foundation of the validity of TTSP lies in a core assumption—that the material possesses Thermorheological Simplicity [23,24], meaning that the molecular relaxation modes or their distribution that controls the material’s viscoelasticity or aging response does not change with temperature; only the relaxation time varies with temperature (following equations such as WLF) [25,26]. Unfortunately, this crucial assumption does not hold in many practical and complex engineering scenarios.

For instance, when polymers are in the glass transition region or undergo structural changes such as partial crystallization or phase separation, the nature of their dominant relaxation modes (such as segmental motion, secondary motion) may change. What is even more complex is that in real service environments, polymers generally endure the combined effects of multiple stress fields, including temperature fluctuations and mechanical stress cycles, wet–heat coupling, or irradiation [27,28]. Under the complex coupling of these stresses, the aging mechanism itself may transform (such as a shift from thermal degradation to stress–chemical degradation), leading to the molecular relaxation spectra at different temperatures no longer having a simple scaling relationship [29,30]. Once the Thermorheological Simplicity assumption is violated, the horizontal and vertical shift factors on which TTSP is based will lose their physical significance or become inapplicable, resulting in the master curves and extrapolation results constructed accordingly deviating significantly from the actual aging trajectory of the material, and greatly reducing the reliability of its predictions.

### 2.3. Numerical Fitting Method

To overcome the limitations of the aforementioned models in specific datasets or complex conditions, numerical fitting methods are often employed to enhance the curve-fitting accuracy of polymer life prediction in particular scenarios [31,32]. Such methods are typically based on polynomial, power-law or custom function forms, and they optimize the approximation of experimental aging data (such as performance degradation curves) by adjusting a large number of parameters [33,34,35].

However, the essence of such methods lies in mathematical optimization and approximation techniques rather than revealing or modeling the intrinsic physical and chemical mechanisms of material aging. Their core shortcomings are twofold: Firstly, the model parameters lack clear physical significance and cannot represent specific chemical reaction pathways, microstructure evolution, or changes in the concentration of free radicals/reactive sites involved in the aging process, thus being typical “black box” or “gray box” models. Secondly, and most critically, these methods struggle to effectively and accurately integrate the nonlinear impacts of other important environmental variables beyond temperature and their interaction effects on the degradation pathways of polymers.

For instance, multiple factors such as ultraviolet radiation dose (or spectral distribution), periodic mechanical stress (amplitude, frequency, mode), environmental humidity, and exposure to chemical media not only may independently lead to unique degradation patterns (such as photo-degradation, fatigue, stress cracking, swelling/dissolution), but also have complex synergistic or antagonistic interactions among themselves and with temperature [13,36,37,38]. These complex nonlinear effects are challenging to accurately characterize and predict using purely data-driven empirical models, particularly when extrapolation extends beyond the range of the experimental conditions.

A comprehensive review of the above-mentioned classic polymer life prediction methods (Arrhenius method, TTSP, and numerical fitting method) clearly reveals their common methodological bottlenecks: one is the oversimplification of the actual physical and chemical essence of polymer aging; the other is the insufficient characterization of the multidimensionality of the service environment. Specifically, these methods tend to simplify the complex aging process, which is essentially driven by the nonlinear synergy and competition of multiple physicochemical mechanisms (such as heat, oxygen, water, light, mechanical force, etc.), into an aging process described by a single dominant variable (mostly temperature) or a mathematical function. This excessive compression neglects the complex interaction effects among environmental stress fields (such as photo-thermal oxidation and wet–thermal degradation).

Furthermore, it fails to fully consider the intrinsic feedback and reshaping effects of the internal microstructure evolution of materials (such as changes in crystallinity, cross-linking/breaking of chains, and phase separation) on the degradation kinetics laws. Meanwhile, the existing methods generally lack effective strategies for real-time assimilation or state updating of the dynamic performance/environment monitoring data that can be obtained during service.

It is precisely due to the inherent limitations of forcibly simplifying and reducing the dimensionality of multi-variable problems, as well as the absence of a dynamic feedback loop between the model and in-service data, that when models constructed based on accelerated aging experiments are extrapolated over extended periods to real-world service conditions—characterized by long periods and complex environments—the uncertainty of the prediction results increases significantly, and model accuracy declines sharply, thereby severely limiting the practical engineering value of the prediction outcomes. Therefore, there is an urgent need to leverage machine learning for developing novel predictive frameworks that effectively integrate multi-mechanism coupling modeling with dynamic, data-driven approaches.

## 3. Machine Learning-Driven Predictive Systems: Frameworks and Applications

A systematic classification of aging modeling approaches reveals two overarching paradigms: traditional methods (e.g., Arrhenius, TTSP, numerical fitting) that rely on simplified assumptions, and machine learning methods that capture multi-mechanism coupling. The latter can be further categorized into: (1) kernel-based models (e.g., SVM) for nonlinear regression in small-sample scenarios; (2) neural networks (e.g., ANN, CNN) for cross-scale data fusion; (3) tree-based models (e.g., RF, XGBoost) for interpretable feature importance quantification; and (4) hybrid models that integrate physical constraints with data-driven intelligence.

Through Table 1, we have listed the limitations of traditional methods and the corresponding advantages of machine learning. Facing the systematic flaws of conventional methods, machine learning models are reshaping the paradigm of polymer lifetime prediction. By constructing intelligent models that integrate data-driven approaches with physical mechanisms, machine learning can not only capture the nonlinear interaction effects of temperature, humidity, environmental stress, and chemical media, but also seamlessly integrate material gene data, accelerated aging maps, and online monitoring signals, establishing a full-chain mapping relationship from molecular motion to macroscopic failure.

### 3.1. Support Vector Machine

In the field of polymer life prediction, support vector machine (SVM), as a machine learning method based on statistical learning theory, achieves regression or classification tasks by constructing the optimal hyperplane in a high-dimensional feature space [39,40]. The core principle is to introduce a kernel function (such as the radial basis function (RBF)) [41,42] to map the non-linearly separable polymer aging data into a high-dimensional space, transform it into a linearly separable problem, and then solve for the global optimal solution based on the principle of structural risk minimization. In the prediction of service life, SVM establishes a mapping model between input variables (features) and remaining life (labels) by analyzing the complex nonlinear relationships among polymer structural characteristics (such as molecular weight distribution, functional group types), environmental parameters (temperature, humidity, ultraviolet irradiation intensity), and dynamic monitoring data (crack propagation rate, dielectric loss factor). By controlling the regression error through the ε-insensitive loss function [43], SVM can output continuous predicted values of the lifespan and utilize the kernel trick to handle the interaction of high-dimensional features, effectively capturing the coupling effects of multiple mechanisms (such as the synergistic acceleration of thermal oxidation and hydrolysis).

#### 3.1.1. Case Analysis of SVM

In response to the challenges posed by the large dispersion in the fatigue life of rubber automotive vibration isolators and the limitations associated with small sample sizes in reliability assessment, Liu et al. [44] conducted uniaxial tensile fatigue tests to obtain life data under varying logarithmic strain peaks (S = 0.405 − 0.916). Using the SVM model—where empirical reliability served as input and fatigue life as output—the sample size was expanded to 100 groups, with optimized RBF kernel parameters (g = 0.71 − 5.66). The expanded dataset was validated to conform to a lognormal distribution via the Kolmogorov–Smirnov (K-S) test, leading to the establishment of a probabilistic stress-life (P-S-N) model. This study successfully overcame the limitations of small sample sizes by employing SVM-based data expansion to address data insufficiency. The K-S test confirmed the validity of the lognormal distribution (S_n_ < 1.358), offering a novel approach for probabilistic modeling. Furthermore, the research enabled the quantification of reliability, as the P-S-N curve illustrated life degradation at different reliability levels (e.g., a 30–40% reduction in life at 90% reliability compared to 50%), thereby supporting engineering safety design with prediction errors remaining within 1.5 times the dispersion range of the measured values.

**Figure 1:**
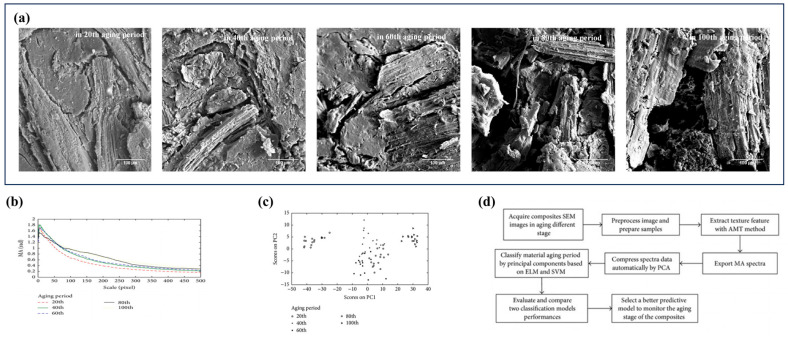
Microstructural evolution and feature analysis of wheat straw/polypropylene composites under accelerated aging. (**a**) Representative SEM images showing progressive crack development. (**b**) MA spectra of SEM image samples of the composites in 5 different aging periods. (**c**) PCA scores plot demonstrating effective feature space separation. (**d**) Schematic workflow of the image analysis and classification pipeline [45].

Zhang et al. [45] investigated the extraction of texture features from SEM images of wheat straw/polypropylene composites during accelerated aging by employing angle measurement technology. Following feature extraction and dimensionality reduction using principal component analysis (PCA), both the extreme learning machine (ELM) and SVM algorithms were applied to achieve intelligent classification of aging stages. Experimental results demonstrated that as the ultraviolet aging cycle increased from 20 to 100 cycles, SEM images revealed a progressive expansion and deepening of surface cracks (Figure 1a), exemplifying classic macroscopic defects such as cracking and delamination associated with photo-aging mechanisms, accompanied by a significant increase in mass spectrometry complexity (Figure 1b). PCA indicated that the first four principal components accounted for 97.4% of the total variation in texture features (Figure 1c). Classification performance showed that the SVM model achieved a higher accuracy (92.5%) compared to the ELM model (86.3%), with SVM demonstrating a sensitivity of 81.3–100% in detecting the mid-term aging stage (40–80 cycles). These findings confirm that the angle measurement technology combined with PCA can effectively quantify the microstructural evolution associated with material aging (Figure 1d), offering a novel, high-precision, non-destructive method for assessing aging in bio-based composites.

Mohanty et al. [46] investigated the oxidative stability of butadiene rubber (BR) with various antioxidants by monitoring changes in Mooney viscosity and color difference index, thereby assessing the impact of antioxidant concentration on BR degradation stability. The data science model effectively simulated the observed variations in Mooney viscosity and antioxidant concentration across different experimental runs, as well as their corresponding levels of color. RFM was utilized to model changes in Mooney viscosity, while SVM was applied to model the measured color difference index based on experimental parameters such as antioxidant concentration, temperature, and sample aging time. Validation through additional experiments further confirmed the accuracy of the Mooney viscosity model.

The application of the above-mentioned SVM in the prediction of polymer lifespan was shown in the Table 2 as follows:

#### 3.1.2. Advantages and Limitations of SVM

The core advantages of SVM:

1. Small Sample Adaptability

Compared to other machine learning models, the SVM demonstrates superior generalization performance when applied to small sample datasets [47]. Polymer long-term aging test data are scarce. For instance, in the works of Zhang et al. [45] and Mohanty et al. [46], the sample sizes involved were 80 groups and 72 groups, respectively, and the data volumes were relatively small. The SVM reduces dependency on large data volumes by leveraging the sparsity of support vectors—relying only on boundary samples—and maintains high predictive stability even with limited datasets.

2. Nonlinear Modeling and Global Optimality

The kernel function can flexibly represent the nonlinear dynamic process in polymer aging, avoiding the simplified assumption of temperature dependence in the traditional Arrhenius equation [48]. Its convex optimization characteristic guarantees the uniqueness and global optimality of the solution, thereby mitigating the issue commonly encountered in neural networks of becoming trapped in local optima.

3. Noise Resistance and Robustness

The complexity of the model is controlled through the regularization parameter (penalty factor C), which makes the model insensitive to data noise and outliers and enhances its robustness [49]. For instance, in the accelerated aging test of polymers (Zhang et al. [45]’s study on wheat straw/polypropylene composites), SVM effectively processed the texture noise in SEM images by optimizing parameters, achieving a classification accuracy as high as 92.5%.

The main limitations of SVM:

1. Data Dependency and Feature Engineering Bottleneck

The performance of SVM is heavily influenced by the effectiveness of feature engineering. Polymer aging encompasses multi-scale mechanisms that span from the molecular to the macroscopic level. These mechanisms necessitate the manual design of specific features—such as crack fractal dimension and the infrared absorption attenuation rate of functional groups—making it challenging to automatically extract deeply correlated features. In contrast, deep learning models are capable of autonomously identifying and learning such latent patterns.

2. Parameter Sensitivity and Tuning Cost

The selection of kernel function type, penalty factor C, and kernel parameters (such as the γ value of RBF) significantly affects the prediction accuracy [50,51]. Traditional grid search parameter tuning has high computational costs, and the differences in polymer multi-batch data require repeated re-tuning, which restricts its engineering practicality.

3. Lack of Explainability

As a “black box” model, SVM cannot directly reveal the dominant aging mechanisms (such as the antioxidant depletion threshold, hydrolysis reaction pathways), making it difficult to guide material modification design [52]. In contrast, models like decision trees can provide rankings of feature importance.

In conclusion, SVM, with its advantages of small sample size and strong generalization ability, provides a feasible tool for polymer life prediction in scenarios with scarce data. However, their future development needs to break through the bottlenecks of feature engineering dependence and mechanism interpretability. This can be achieved by embedding physical constraints, cross-model fusion, and adaptation to edge computing, promoting a paradigm shift from “data-driven” to “mechanism-data dual-driven”.

### 3.2. Neural Network Model

In the field of polymer lifetime prediction, neural networks have emerged as a critical technology for overcoming the limitations of traditional approaches, owing to their robust capabilities in nonlinear modeling. The fundamental advantage of these models lies in their capacity to emulate the human brain’s mechanisms for processing information, enabling the construction of multi-layered computational architectures that can autonomously extract material aging patterns from complex and high-dimensional datasets [53,54]. This facilitates an accurate mapping of the remaining useful life of polymers. The operational principle can be summarized as follows: The input layer collects various environmental parameters, such as temperature, humidity, and stress amplitude, as well as material properties, including molecular weight distribution and crosslinking density. The hidden layer progressively abstracts hierarchical features through nonlinear activation functions, such as Sigmoid and ReLU. Finally, the output layer performs regression to predict the material’s lifespan [55,56].

#### 3.2.1. Artificial Neural Network

Artificial neural networks/backpropagation neural networks (ANN/BPNN), process structured data (such as temperature and humidity time series, chemical components) through a fully connected structure and excel at capturing the coupling effects of multiple variables (such as the synergistic acceleration of hydrolysis by wet heat). During the training process, the BPNN adjusts the connection weights between neurons based on the gradient of the prediction error, enabling the model to continuously approach the true physical laws of the material aging process [57,58]. However, ANN/BPNN models are prone to getting stuck in local optimal solutions and rely on manually designed features, making it difficult to automatically extract the cross-scale correlations between molecular structures and macroscopic properties.

**Figure 2:**
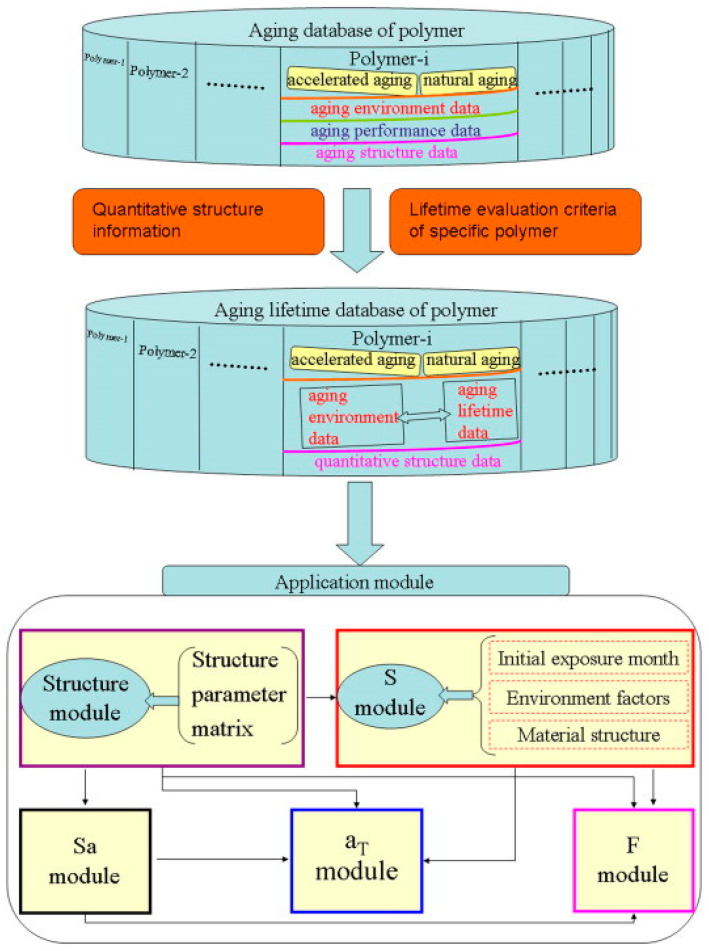
Diagram of polymer aging life prediction system [59]. Reproduced with permission from Han Liu, Mingyong Zhou, Yuli Zhou, Shan Wang, Guangxian Li, Long Jiang, Yi Dan, Polymer Degradation and Stability; published by Elsevier, 2014.

The application of artificial neural networks in predicting the aging lifespan of polymer materials was initially proposed by Liu et al. [59] The specific approach is illustrated in Figure 2, which entailed the establishment of two standards: one for quantifying structural information and another for assessing the life of specific polymer materials, to effectively manage data from the polymer material aging database. A data processing module was developed for relevant data, where the Structure module was utilized to transform the polymer material’s structure into a matrix of structural parameters. Conversely, the S module employed three components—initial exposure months, environmental factors, and material structure—to accurately calculate the level of environmental stress. The Sa module was utilized for stress level computation under accelerated environmental conditions. The a_T_ module investigated the correlation between specific accelerated aging tests and the distribution of life in natural aging tests, while the F module generated characteristics of aging life distribution. The method was employed to investigate the distribution of aging life within a specific polycarbonate aging area, as well as the corresponding relationship between accelerated aging tests and outdoor exposure test life distributions. The time error fell within 15 days (aging total time within 7 months), indicating the model’s high accuracy. This study established a solid foundation for practical applications and effectively showcased the immense potential of artificial neural networks in predicting the aging life of polymer materials.

**Figure 3:**
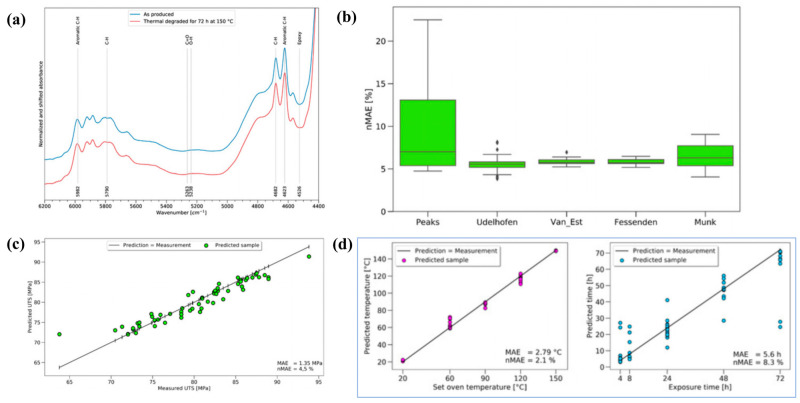
FTIR-ANN approach for predicting thermal aging of epoxy resin. (**a**) Key FTIR spectral bands in the NIR region. (**b**) Optimization of data preprocessing algorithms. (**c**) Correlation between predicted and measured UTS. (**d**) Model prediction accuracy for temperature and exposure time [60].

Audrius Doblies and colleagues [60] proposed an innovative approach that integrated FTIR with ANN to predict the thermal exposure behavior and residual mechanical strength of epoxy resin. Samples of varying thicknesses were prepared to systematically investigate the chemical and mechanical alterations induced by thermal exposure at temperatures ranging from 60 to 150 °C and exposure durations of 4 to 72 h. FTIR analysis revealed oxidation, chain scission, and dehydration in thin film samples, whereas near-infrared spectroscopy exhibited limited sensitivity to chemical changes in bulk materials (Figure 3a). Through data preprocessing and optimization of feature extraction algorithms (Figure 3b), the ANN model effectively extracted meaningful features from highly variable mechanical data and subtle spectral variations. As a result, the model achieved a residual strength prediction error of only 1.35 MPa (NMAE = 4.51%), with temperature and time prediction errors of 2.1% and 8.3%, respectively (Figure 3c,d). These results demonstrate the high sensitivity of the FTIR-ANN approach in detecting microscopic damage in bulk materials.

**Figure 4:**
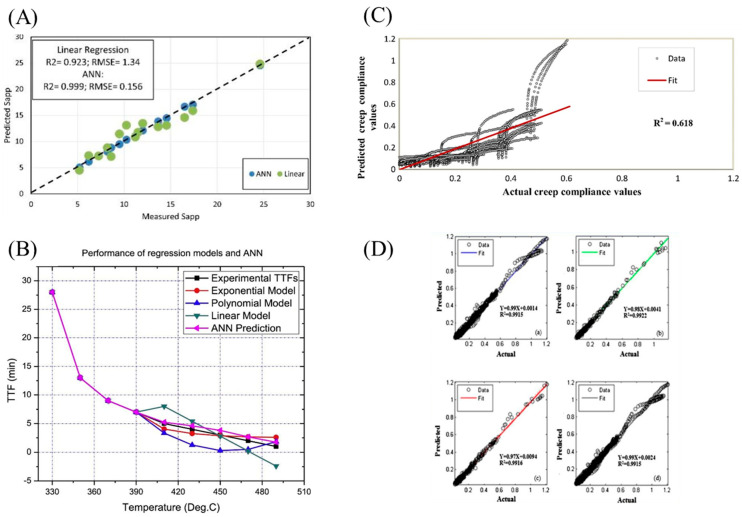
(**A**) The fitting effect of ANN compared to linear regression models [61]. (**B**) Comparison between linear regression models and ANN output layer and experiments [62]. (**C**,**D**(**a**–**d**)) High accuracy of ANN in predicting creep compliance of asphalt mixtures across training, validation, and testing datasets [63].

The ANN significantly reduced the time and effort required for conducting artificial aging experiments on polymer materials while demonstrating a more pronounced advantage in terms of accuracy when compared to numerical fitting methods. Zhang et al. [61] used the real-time state and parameters of adhesive and mixture cracking tests to study the variation in the fatigue performance of asphalt binder and mixture between different mixture designs and evaluate their aging conditions over time. They developed a predictive model for estimating the fatigue performance of asphalt mixtures based on linear fitting and ANN, which only required material design information and adhesive aging conditions as inputs to predict aging life. As shown in Figure 4A, compared to linear fitting, the ANN model exhibited higher R^2^ values and lower RMSE values, demonstrating better fitting performance. This improvement stemmed from the ANN’s ability to automatically capture nonlinear interactions between aging factors through its hidden layers while adaptively weighting input features, thereby resolving the oversimplification of Arrhenius-type linear assumptions.

Furthermore, Santhosh et al. [62] employed a comprehensive approach combining numerical fitting and ANN to predict the life and reliability of industrial control cables. These predictions were made based on models derived from fitting the entire experimental data set. The comparison results between numerical fitting, ANN, and experimental data in Figure 4B show that ANN prediction was able to capture trends in cable aging processes with limited training data availability, closely resembling the trends observed in experimental data.

Moreover, Alrashydah et al. [63] proposed two prediction models, namely the nonlinear fitting model and ANN, to forecast the creep compliance performance of asphalt mixtures. These models were evaluated based on factors such as loading time, test temperature, asphalt modification, void level, and aging conditions. As depicted in Figure 4C,D, it was evident that both models can effectively predict the creep behavior of enhanced asphalt mixtures. However, compared to the nonlinear fitting model, which only explained 61% of the measured data, the ANN demonstrated a remarkable ability to explain over 99% of the measured data. Furthermore, with a lower RMSE value than that of the nonlinear fitting model, it exhibited superior accuracy. Zhang et al. [64] conducted an aging experiment on PMMA under ultraviolet light conditions and developed a life prediction model for PMMA using nonlinear curve fitting and ANN. Through comparison of the goodness of fit, simulation, and modeling capabilities with the initial data, as well as the predictive ability for new data, it was observed that the ANN prediction model outperformed the nonlinear curve fitting model. In this study, utilizing the yellowing index of PMMA as an indicator, the output data from the ANN prediction model were used to estimate a projected life of 7.83, 8.47, and 8.42 years for newly produced PMMA samples.

The application of the above-mentioned ANN Models in the prediction of polymer lifespan was shown in the Table 3 as follows:

#### 3.2.2. Convolutional Neural Network

Convolutional Neural Network (CNN) is a type of feedforward neural network that automatically extracts local features of input data (such as images and voices) through convolutional layers and pooling layers. It is widely used in tasks such as visual recognition. The core advantage of CNN lies in its ability to extract spatial features [65,66]. The convolutional kernel model effectively identifies micro-morphological patterns through local receptive fields and parameter sharing, transforming visual data into quantifiable biological indicators. However, this approach primarily captures local characteristics and is limited in its capacity to model long-term temporal dependencies.

Li et al. [67] proposed a CNN-based method for predicting the fatigue life of rubber materials, which overcame the limitation of traditional physical models in handling the coupling effects of multiple parameters by integrating multiple factors such as environmental temperature, material hardness, and peak engineering strain as input. The experiments utilized dumbbell-shaped natural rubber specimens and conducted uniaxial tensile fatigue tests at temperatures of 23 °C, 60 °C, and 90 °C, resulting in a dataset comprising 24 working conditions. The CNN model achieved a training set accuracy of 99.08% and a test set accuracy of 96.14%, significantly outperforming traditional physical models, BPNN, and SVM. All predicted values fell within the 1.5-time discrete band of the experimental values, meeting the engineering accuracy requirements. Compared with BPNN (where some predicted values exceeded the 2-time discrete band) and SVM (where some exceeded the 1.5-time discrete band), CNN demonstrated stronger adaptability and stability with small samples, and its advantage originated from the feature extraction ability of the convolutional layer for the nonlinear relationships of multiple factors. This study provided a high-precision data-driven solution for the durability design of rubber vibration isolators (such as powertrain mounts and torsional vibration dampers), verifying the universal advantage of CNN in predicting the fatigue of rubber under complex working conditions.

Meng et al. [68] proposed a convolutional neural network (CNN)-based method for predicting the service life of epoxy coatings subjected to rapid failure in deep-sea environments. A comprehensive failure model based on the “performance-structure” relationship was established, enabling the CNN to achieve accurate detection of surface cracks in the coatings. Specifically, they constructed a customized CNN (including convolutional layers, pooling layers, and fully connected layers), combined with post-processing techniques (such as Sobel edge detection and Hough transform), to identify crack regions in SEM images. This CNN model was trained using 640 × 640-pixel input images, achieving an overall accuracy of 82.81%, as validated by the ROC curve and confusion matrix. It effectively quantified the crack length distribution, including key metrics such as the L50 value. The extracted crack data were further transformed into kinetic equations, such as the exponential growth model, to characterize the degradation behavior of the coating structure. In conjunction with performance parameters, including adhesion (modeled using the GM(1,1) model) and water absorption rate (described by the Case II diffusion equation), the three primary influencing factors were analyzed using grey relational analysis. The crack factor was assigned a weight of 0.3321, reflecting its significant impact. A life prediction model was subsequently established based on the integrated weights. The model achieved an average prediction error of only 2.60%, demonstrating that CNN-driven image recognition could effectively capture microstructural degradation dynamics and provide a novel framework for coating life prediction.

**Figure 5:**
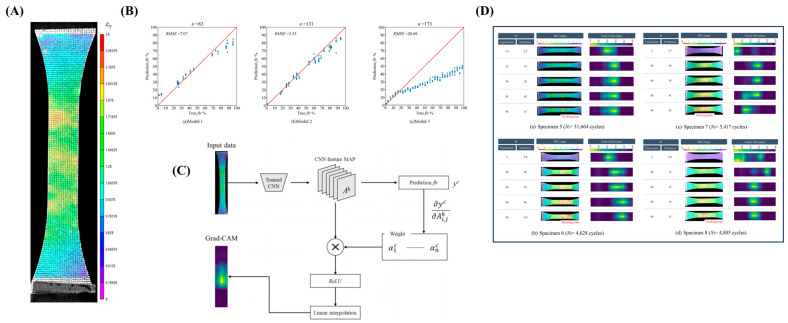
CNN-based fatigue life prediction using DIC and explainable AI. (**A**) Representative DIC strain distribution map. (**B**) Model prediction results with low RMSE. (**C**) Grad-CAM architecture for interpretability. (**D**) Progression of strain localization and model-attention regions leading to failure [69].

Yuta Mizuno et al. [69] innovatively integrated digital image correlation (DIC) with a CNN based on the Xception architecture to predict the fatigue life of short fiber-reinforced plastics. By capturing surface strain distribution images (Figure 5A), the authors developed a regression model that outputs the fatigue life ratio (flr). The model achieved relatively high prediction accuracy when trained and tested on samples from the same batch; however, its generalization capability was limited in cross-sample predictions (Figure 5B), suggesting a sensitivity to individual material variations. To interpret the model, gradient-weighted class activation mapping (Grad-CAM) was employed to visualize the regions of interest within the images (Figure 5C). The results of Figure 5D revealed that the model could identify the fracture initiation site at an early stage of fatigue (flr = 20–40%), significantly earlier than human visual assessment (flr = 60–70%), demonstrating the ability of CNN to detect subtle, early-stage damage features. This approach presented a novel framework for early material failure diagnosis using image-based recognition techniques; however, further improvements in cross-sample generalization were necessary, which could be achieved by expanding the diversity of the training dataset.

Zhang et al. [70] innovatively developed an automatic detection system for the degradation mechanism of photovoltaic backsheet based on a fully CNN, which achieved pixel-level recognition of six types of cracks (parallel, mudcrack, delamination, etc.) through end-to-end semantic segmentation. To address the challenge posed by the limited sample size (only 34 original images), block segmentation and mirror augmentation were implemented to expand the dataset to a total of 286 samples. Additionally, the class imbalance issue related to crack detection was mitigated through the adjustment of class weights, employing a weighted cross-entropy loss function. The network architecture adopted a 16-layer convolutional encoder and a feature fusion decoder, combined with Gaussian Dropout (λ = 0.5) and L2 regularization to suppress overfitting. Experimental verification showed that this method achieved 92.8% pixel accuracy and 72.5% mean intersection over union in both accelerated aging and real environment data. It only took 2.1 s for single-image detection, significantly outperforming the efficiency of traditional manual inspection, providing an automated solution for the operation and maintenance of photovoltaic power stations.

The application of the above-mentioned CNN Models in the prediction of polymer lifespan was shown in the Table 4 as follows:

Furthermore, a comparative analysis between CNN and ANN highlights their distinct applicability depending on data modality and prediction tasks. While ANN models excel in processing structured data (e.g., time series of environmental parameters or scalar material properties) and capturing complex nonlinear interactions through fully connected architectures, CNN models demonstrate superior performance in handling spatial and image-based data. For instance, in the crack detection and microstructure analysis of epoxy coatings [68] and photovoltaic backsheets [70], CNN’s inherent capabilities in local feature extraction and translation invariance enabled direct interpretation of SEM images, achieving pixel-level accuracy above 92.8%. In contrast, ANN would require extensive manual feature engineering to process the same image data, potentially introducing subjectivity and losing spatial context. However, ANN retains advantages in scenarios requiring integration of heterogeneous, non-spatial data streams. For example, Doblies et al. [60] successfully combined FTIR spectra and mechanical properties using ANN to predict thermal aging of epoxy resins, a task less suited for standard CNN architectures. Thus, the selection between CNN and ANN should be guided by data characteristics: CNN is optimal for image-centric tasks, leveraging its inductive biases for spatial hierarchies, whereas ANN offers flexibility for fusing diverse tabular and sensor data in multi-mechanism aging models.

#### 3.2.3. Advantages and Limitations of Neural Network Models

The core advantages of neural network models [71,72]:

1. Nonlinear Modeling: Deep hidden layers are capable of capturing complex dynamical behaviors that are challenging to characterize using traditional mathematical equations—for instance, the abrupt changes in activation energy observed during the glass transition phase.
2. Multi-source data fusion: integration of physical model outputs, in situ monitoring signals (including dielectric spectroscopy and acoustic emission), and material genome data, to establish a multi-scale predictive framework.
3. Dynamic extrapolation capability: The system enables real-time updating of weights to effectively adapt to sudden environmental changes, such as abrupt variations in temperature and humidity.

However, its inherent limitations should not be overlooked [71,72]:

1. Data dependency: Deep neural networks necessitate a substantial amount of labeled data for effective training. However, long-term aging data for polymers are limited, which increases the risk of overfitting.
2. Limited physical interpretability: The black-box characteristic of the model impedes the analysis of key aging mechanisms (e.g., the threshold for chain scission reactions), thereby complicating the guidance of material modification strategies.
3. Insufficient cross-condition generalization: New material formulations or exposure to extreme environments (e.g., combined irradiation and stress) may lead to a failure in predictive performance.

The role of neural networks in predicting polymer lifetimes is transitioning from a “data-driven tool” to a “mechanism-data integrated engine.” In the future, through the endogenous integration of physical constraints, cross-scale dynamic modeling, and edge-based lightweight deployment, it is anticipated that a new generation of intelligent predictive paradigms will emerge. These paradigms will combine high prediction accuracy, mechanism interpretability, and engineering applicability, thereby providing fundamental support for the full life cycle management of polymers.

### 3.3. Decision Tree Model

In the field of polymer life prediction, decision tree models (including random forest and XGBoost, etc.) provide a unique solution for modeling material aging behavior by constructing multi-level regularized decision paths. The core principle involves decomposing the complex material degradation process into quantifiable feature-splitting logic. By leveraging ensemble learning mechanisms, algorithms such as Random Forest and XGBoost aggregate the prediction results of multiple decision trees, thereby enhancing the model’s robustness and generalization capability [73,74,75]. For example, when predicting the thermal oxidative aging life of polymers, the model may initially segment the data based on temperature thresholds, subsequently refining the decision branches by considering oxidation induction time or carbonyl index. The final estimated life values are then generated at the terminal nodes. This hierarchical decision-making framework, grounded in feature importance, offers a visual representation of the nonlinear degradation pathways of polymers.

#### 3.3.1. Random Forest Model

The Random Forest Model (RFM) [76,77] is a highly representative parallel ensemble method that employs decision trees as base classifiers to address the issue of overfitting commonly encountered in decision trees. The RFM incorporates two mechanisms of randomization during the process of constructing the forest. The training samples for constructing a single decision tree are extracted consecutively using the random sampling technique. At each node of the tree, a random feature subspace is selected to determine the attribute for splitting and building the tree. Finally, by employing majority voting, the prediction result of the RFM is obtained by combining the results of individual decision trees. The random forest model effectively addresses the performance bottleneck issue encountered by decision trees and exhibits excellent compatibility with noise, rendering it extensively employed in the domain of material science.

Based on experimental data of rheological properties, Salehi et al. [78] developed the RFM to accurately predict the complex shear modulus and phase angle of Recycled Plastic Modified Bitumen under non-aged and short-term conditions. The predictive performance of RFM was evaluated using metrics such as MAE, RMSE, R^2^, and objective function value. The results demonstrated that RFM exhibited exceptional accuracy with R^2^ values of 0.98 and 0.93 for predicting the complex shear modulus and phase angle, respectively. To investigate the atmospheric aging phenomenon of acrylic coatings, Li et al. [79] conducted a two-year exposure experiment in 13 representative climatic environments across China. Based on the aging data collected from 567 cities and considering 11 environmental factors, an atmospheric aging evaluation model for acrylic coatings was established. The data underwent processing using a combination of RFM and Spearman correlation analysis to reduce redundancy and multicollinearity. Subsequently, a semi-supervised co-training regression model was developed with the low-frequency impedance modulus value obtained from electrochemical impedance spectroscopy of acrylic ester coatings in a 3.5wt% NaCl solution as the output variable, while environmental factors were used as input variables. This novel approach provides a rapid method for evaluating the aging performance of acrylic coatings and serves as a valuable reference for assessing the aging behavior of other organic coatings.

**Figure 6:**
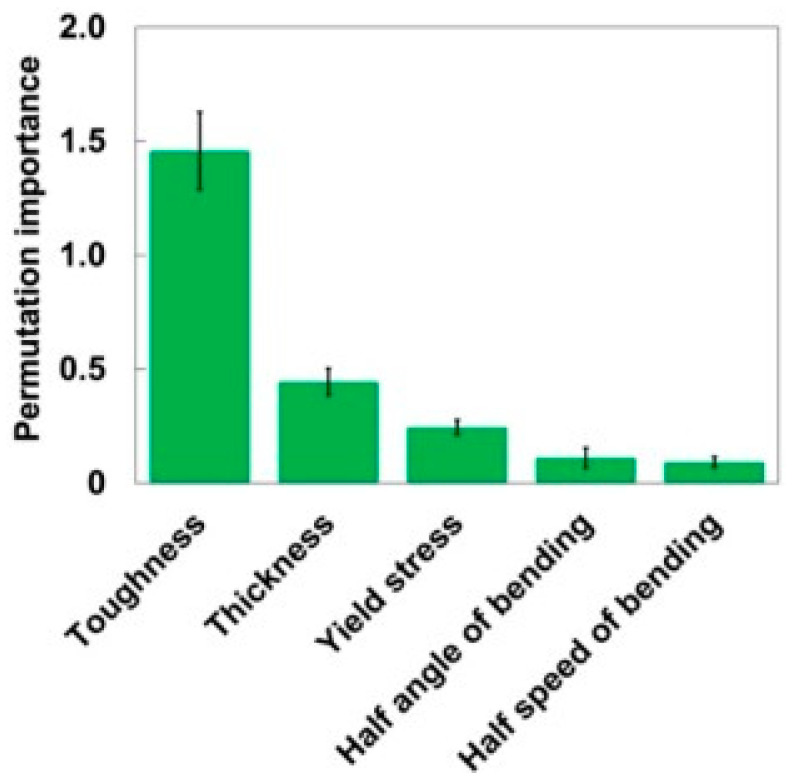
Permutation importance of each feature [80]. Reproduced with permission from Masayuki Kishino, Kohsuke Matsumoto, Yoshiaki Kobayashi, Ryo Taguchi, Norihisa Akamatsu, Atsushi Shishido, International Journal of Fatigue; published by Elsevier, 2023.

To address the bending fatigue challenges associated with polymer films in flexible electronic devices, Masayuki Kishino and his team [80] initially conducted fatigue tests to collect 152 sets of fatigue life data across six materials (COP, PC, PVC, PET, PEN, and PS) under varying conditions, including thicknesses ranging from 100 to 300 μm, bending half-angles (θ/2) between 60° and 90°, and bending half-speeds (v/2) from 60 to 360 °/s. Subsequently, they developed a machine learning model by selecting relevant features—identifying five key input parameters such as toughness, thickness, and yield stress while eliminating redundant variables like Young’s modulus—and optimizing the Random Forest Model (RFM) hyperparameters (depth = 15, number of trees = 50) using grid search. The model was then trained to predict fatigue life. The key contributions of this study are twofold. First, it offered an efficient and accurate predictive capability, with the RFM achieving a mean absolute percentage error (MAPE) of 22.3% and an R^2^ value of 0.892 on the test set, along with a computation time of less than one minute. These results significantly outperformed those of linear regression, thereby providing a rapid and reliable tool for selecting flexible substrates. Second, the study introduced interpretability innovations by identifying critical influencing factors—toughness, thickness, and yield stress—as revealed through feature importance analysis (Figure 6), offering valuable insights for material design and optimization.

#### 3.3.2. XGBoost Model

Extreme Gradient Boosting (XGBoost), as an efficient implementation of the gradient boosting framework, derives its core performance advantages from the integration of three coordinated optimization techniques [73,81]. First, it incorporates a regularized objective function by explicitly introducing penalty terms for the number of leaf nodes (γ) and the weight norm (λ/α) into the loss function. This approach effectively constrains structural complexity during the model training phase, thereby significantly reducing the risk of overfitting [82,83]. Second, XGBoost introduces a second-order Taylor expansion-based acceleration mechanism, which overcomes the limitation of traditional Gradient Boosted Decision Trees that rely solely on first-order gradient information. By simultaneously utilizing both first-order derivatives (gradients) and second-order derivatives (Hessian matrices), the algorithm achieves a more accurate approximation of residuals, enhancing the precision of split point computation and accelerating convergence [84,85].

Furthermore, XGBoost features a sparse-aware and parallelization-oriented design, which provides engineering-level advantages. It automatically learns the optimal split direction for missing values, eliminating the need for manual imputation. Additionally, techniques such as feature pre-sorting and bucketing enable parallel computation of split gains, allowing for efficient processing of high-dimensional data. These three optimization strategies work synergistically across the dimensions of generalization, accuracy, speed, and engineering efficiency, ultimately enabling XGBoost to deliver significant performance advantages in handling large-scale datasets within industrial applications.

**Figure 7:**
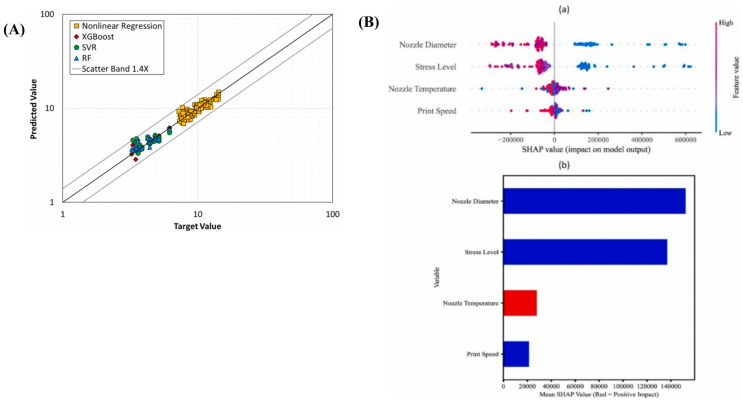
(**A**) The scatter-band analysis of different models. (**B**) ML prediction results: (**a**) SHAP bee swarm and (**b**) bar plots [86]. Reproduced with permission from Hamid Nasiri, Ali Dadashi, Mohammad Azadi, Materials Today Communications; published by Elsevier, 2024.

Hamid Nasiri et al. [86] proposed a fatigue life prediction methodology for 3D-printed polylactic acid (PLA) biomaterials by leveraging interpretable machine learning techniques. The approach integrated the XGBoost algorithm with the Shapley Additive explanations (SHAP) interpretability framework, enabling accurate and transparent fatigue life prediction. The experimental dataset was derived from 115 groups of standardized dog-bone-shaped specimens, encompassing critical printing parameters such as nozzle diameter (0.2–0.6 mm), printing speed (5–15 mm/s), nozzle temperature (180–240 °C), and applied stress level (2.5–17.5 MPa). Fatigue testing was conducted using a rotating bending fatigue testing system operating at a frequency of 100 Hz. Comparative analysis (Figure 7A) demonstrated that the XGBoost model significantly outperformed alternative models, including random forest (R^2^ = 94.30%), support vector regression (R^2^ = 44.88%), and conventional nonlinear regression (R^2^ = 81.20%), achieving an R^2^ value of 97.66 with a narrow prediction dispersion band of ±1.3. This superior performance can be attributed to the regularization mechanisms and the design of the loss function based on second-order Taylor expansion, which effectively mitigates overfitting risks. SHAP-based interpretability analysis (Figure 7B) identified nozzle diameter (negatively correlated) as the most influential parameter, followed by stress level and nozzle temperature, whereas printing speed exhibited the least impact. Notably, this conclusion diverges from the “stress level dominance” observed in traditional regression analysis, underscoring the intricate interactions among process parameters. This study represents the first application of interpretable machine learning in the fatigue behavior analysis of 3D-printed biomaterials, offering a data-driven framework for optimizing printing parameters and enhancing the durability of PLA-based components. Furthermore, it confirms the robustness and general applicability of XGBoost in modeling complex process–property relationships.

Lu et al. [87] proposed a strategy for predicting the contact fatigue life of polymer gears based on a hybrid data-driven approach of experiments and simulations. By integrating Conditional Table GAN data augmentation with the XGBoost algorithm, they achieved highly accurate life predictions. The experiment utilized polymer gears made of materials such as PEEK, POM, and POK, with a module of 1 mm and a tooth width ranging from 20 to 22 mm. Contact fatigue tests were conducted under both oil lubrication (at temperatures between 30 and 80 °C) and grease lubrication conditions. The failure criterion was established as a pitting area on the gear tooth surface exceeding 10%. The numerical model was established based on the thermo-visco-elastic–plastic constitutive relation and the modified Brown–Miller multiaxial fatigue criterion. The gear meshing process was simulated by Abaqus, and the consistency of the contact stress with the standard was verified. The mixed data, which combined experimental and simulation data in a 1:3 ratio (40 groups), was augmented to 10,000 samples using Conditional Table GAN and then input into the XGBoost model. The prediction accuracy of this model within a 3-fold discrete band reached 100%, which was significantly better than that of traditional RFM, SVM, and unoptimized models. SHAP analysis indicated that the maximum contact stress (with a contribution of 48.3%), elastic modulus (19.8%), and tensile strength (14.7%) were the primary influencing factors, whereas the coefficient of friction and Poisson’s ratio exhibited minimal impact. Based on these findings, a formula for estimating the engineering service life was developed. The prediction error of this formula remained within a fivefold dispersion range, offering a robust and reliable reference for the design of polymer gears in high-load applications, such as aero engines and new energy vehicles.

The application of the above-mentioned Decision Tree-Based Models in the prediction of polymer lifespan was shown in the Table 5 as follows:

#### 3.3.3. Advantages and Limitations of the Decision Tree Model

The core advantages of the decision tree model lie in its strong interpretability and broad adaptability in engineering applications, as detailed below:

1. Quantification of Feature Importance: By calculating the information gain resulting from feature splitting, the primary factors influencing lifespan can be clearly identified, thereby providing a well-defined direction for material optimization [88].
2. Adaptability to small samples and imbalanced data: Its insensitivity to missing values and outliers enables it to effectively handle sparse datasets in polymer accelerated aging tests [89].
3. Multi-mechanism coupling modeling: The feature random subspace sampling (max_features) employs in random forest is capable of capturing the interactive effects among temperature, stress, and chemical corrosion, thereby avoiding the oversimplification associated with relying on a single dominant mechanism, as is commonly done in the traditional Arrhenius equation [90].

However, its inherent limitations also restrict its application in complex scenarios:

1. Overfitting risk: A single decision tree is prone to noise interference and generates overly complex structures, leading to excessive adaptation to the training data. This may result in the misjudgment of material defects in a specific batch as universal rules [91,92].
2. Defects in the processing of continuous variables: The threshold-based splitting method is inadequate for accurately capturing the gradual influence of continuous variables, such as activation energy and diffusion coefficient. It may also fail to account for nonlinear transitional behaviors near the glass transition region [93,94].
3. The absence of cross-scale correlation: It is challenging to establish an inherent link between molecular structure and macroscopic properties without external intervention, thereby necessitating reliance on manual feature engineering.

### 3.4. Hybrid and Integrated Model

#### 3.4.1. Integrated Architecture Innovation: Material Potential of Deep Stacking and Chain Integration

The deep stacked architecture first extracts primary features via base learners and subsequently captures the nonlinear degradation effects through meta-learners, making it well-suited for the collaborative prediction of thermal oxidative aging and stress relaxation in polymer materials [95,96]. Chained integration establishes a sequential connection between physical models and data-driven models. For example, one can first simulate the mobility of polymer chain segments using molecular dynamics and subsequently utilize the results as input for a Long Short-Term Memory (LSTM) model to predict long-term creep failure. This architectural approach effectively combines the interpretability of mechanistic models with the capability of data models to capture complex, nonlinear relationships. However, the reliability of this method may be compromised by error propagation from the base models, as well as the limited availability of accelerated aging data for polymers. Meanwhile, a residual correction module can be incorporated into the system. By integrating digital twin technology, a virtual model capable of simulating multi-mechanism coupling aging processes in polymers can be established, thereby generating synthetic data for training the neural network architecture. Furthermore, the quantum annealing algorithm can be applied to improve the computational efficiency of molecular dynamics simulations.

Based on the hierarchical Bayesian meta-modeling framework, Tao et al. [97] investigated high-precision fatigue life prediction for glass fiber-reinforced polymer (GFRP) laminates by integrating physical mechanisms with data-driven approaches. The proposed method established a stacked architecture comprising five damage sub-models (S1–S5), where S1–S3 described matrix crack evolution using shear lag theory and Paris’ law, while S4–S5 incorporated independent delamination damage terms. By dynamically weighting and integrating the outputs of these sub-models through Bayesian stacking, the model effectively addressed the challenge of coupling multiple damage modes in composite materials. The integration of physical equations with sequential Monte Carlo (SMC) inference formed a chained modeling structure: the former established the physical relationship between Lamb wave velocity and damage variables, while the latter enabled updating of the parameter probability distributions. To address the issue of limited sample data, a confidence interval-based criterion (CI-based criterion) was employed to quantify prediction uncertainty, and a prior transfer mechanism was introduced to significantly improve the reliability of early-stage predictions, achieving a prediction error of less than 15% under a 45% stress level. This framework offered an extensible interface for multi-mechanism coupling aging studies of polymer materials, enabling the generation of virtual data via digital twins to support model training. Furthermore, its efficient SMC sampling mechanism provided a compatible pathway for computational acceleration techniques such as quantum annealing algorithms.

#### 3.4.2. Fusion of Probability and Generative Models: Uncertainty Quantification and Small Sample Learning

Probability Ensemble [98,99] is a model fusion approach that leverages probability density functions. Its core principle involves combining the output distributions of multiple base models through weighted averaging, aiming to quantify the overall system uncertainty. This method enhances the robustness of risk assessment through the following mechanism. Firstly, it simultaneously characterizes cognitive uncertainty, which arises from prediction bias due to insufficient model data, and aleatory uncertainty, which represents the inherent randomness of the system. It then employs probability density functions to dynamically assign weights to the outputs of the base model, thereby generating a fused probability distribution. Secondly, by leveraging anchoring integration technology, physical constraints or domain-specific priors are incorporated into base models—such as Bayesian neural networks or Gaussian processes—in the form of regularization. This ensures that the resulting fusion outcomes adhere to fundamental physical laws. The third approach involves performing probability distribution weighting in the output space—for example, by minimizing the Kullback–Leibler divergence—rather than relying on simple result-level voting. This helps maintain probabilistic consistency during uncertainty propagation.

For instance, Lian et al. [100] proposed a physics-data-driven framework for the probabilistic prediction of fatigue life in composite materials, innovatively integrating a stochastic stiffness degradation model with a Bayesian optimization algorithm. A non-normalized stochastic stiffness degradation model (incorporating parameters A, B, c_0_, c_f, etc.) was established to quantify the influence of material uncertainty on fatigue damage accumulation. By employing Bayesian optimization, the fatigue life prediction problem was formulated as a global optimization task within a high-dimensional parameter space, where the objective function was minimized based on stiffness monitoring data, thereby enabling accurate life extrapolation under conditions of limited sample size. Experimental validation demonstrated that, using only 30% to 99% of the available stiffness data as input, 95.64% of the predicted fatigue life values fell within four times the measured values across 102 laminated plate specimens. This approach significantly enhances the engineering applicability of the traditional P-S-N curve method.

Generative adversarial networks (GAN) provide an effective solution to the challenge of limited sample data in material aging detection by synthesizing high-quality microscopic images representing various aging stages, including cracks and voids [101,102]. Its core advantage lies in the direct output of confidence intervals, which supports reliability design and effectively addresses the bottleneck caused by insufficient long-term experimental data [103]. However, the generated data may not conform to physical laws (e.g., violations of the diffusion equation). Therefore, it is essential to incorporate physical constraints into the model optimization process to ensure the plausibility and authenticity of the synthesized data.

Olesja Starkova and her colleagues [104] conducted a systematic review of life prediction models for polymer composites under environmental aging conditions, with particular emphasis on the innovative application of GAN. In addressing the limitations of traditional methods that depend heavily on extensive experimental data, GAN utilized a generator-discriminator adversarial framework to synthesize high-quality virtual datasets, thereby effectively addressing the challenge of modeling with limited samples. When validated against 64 sets of experimental data, the GAN demonstrated a significantly lower average training error (0.28) compared to the twin neural network (0.60). Furthermore, the prediction accuracy, measured by the coefficient of determination (R^2^ > 0.98) and the root mean square error (RMSE = 0.992), approached the theoretical optimum. By integrating wavelet transform (WT) denoising and kernel principal component analysis (kPCA), a hybrid WT-kPCA-GAN model was developed, achieving an average porosity prediction error of only 2.6%. This approach offered an efficient and cost-effective engineering solution for assessing the service life of composite materials, including thermal barrier coatings.

Ye et al.’s study [105] addressed the challenge of assessing thermal barrier coating porosity in scenarios involving limited sample sizes by proposing a life prediction approach based on terahertz non-destructive testing and GAN. By analyzing the coating’s microstructure through scanning electron microscopy and employing a four-parameter random growth method to reconstruct the pore structure, the authors simulated noisy terahertz signals using the finite-difference time-domain method. To overcome the issue of insufficient training data, they innovatively incorporated a GAN framework, in which the generator and discriminator collaborated through adversarial training to produce high-quality synthetic data, thereby significantly improving the model’s generalization performance. In eight-fold cross-validation, the GAN-based model demonstrated superior predictive accuracy compared to the Siamese neural network: the training error was reduced by 53% (average 0.28 vs. 0.60), the coefficient of determination (R^2^) reached 0.98, and key error metrics—root mean square error (RMSE = 0.992) and mean absolute error (MAE = 0.803)—approached their theoretical optimal values. The proposed hybrid model, WT-kPCA-GAN, achieved an average porosity prediction accuracy of 98%, offering an efficient and non-invasive solution for evaluating the service life of thermal barrier coatings in engineering applications.

#### 3.4.3. Hybrid Model of Physical Information: Deep Coupling of Mechanism and Data

The physical-information hybrid model realizes an innovative integration of physical principles and data-driven intelligence through a bidirectional embedding mechanism. At the mechanistic embedding level, the model integrates physical equations—such as mechanical constitutive relations and thermodynamic constraints—as prior knowledge into the neural network architecture. For example, in Physics-Informed Neural Networks (PINN), mechanical equation constraints are enforced through LSTM pathways, while in Conditional Neural Networks (CondNN), the strain energy function is embedded to ensure thermodynamic consistency. This approach compels the model’s output to conform to fundamental physical laws and inherently mitigates the risk of “black-box distortion” commonly associated with purely data-driven methods [106,107].

At the data-driven level, neural networks can learn complex nonlinear relationships that are not captured by mechanistic models through the use of large-scale training data (e.g., the evolution of microscopic defects in materials or responses to sudden environmental changes), thereby effectively compensating for the simplification limitations inherent in traditional analytical approaches. This deep coupling establishes a collaborative framework characterized by “mechanism anchoring direction + data optimizing details”.

On one hand, this approach substantially enhances the model’s extrapolation capability under extreme conditions—such as previously unencountered temperature load combinations. On the other hand, it significantly reduces the demand for data, for example, requiring only 20% of early-stage data to predict the full life cycle behavior. This approach not only overcomes the heavy reliance of traditional methods on costly experimental data but also offers a solution that is both theoretically sound and economically viable for polymer life assessment across multiple scenarios.

In the prediction of the fatigue life of asphalt mixtures, the traditional VECD method faces significant challenges, including high sensitivity to temperature and loading conditions, as well as high experimental costs associated with requiring a 50% modulus reduction. To address these limitations, Han et al. [108] proposed a physically constrained dual-path PINN model that introduces three key innovations. This model incorporated a shallow LSTM pathway that strictly adhered to the VECD mechanical equation constraints, and a deep multi-head attention network designed to capture complex nonlinear distributions. The outputs from the two pathways were integrated through a physical loss function, effectively resolving the “black box distortion” issue commonly associated with traditional artificial neural networks. Furthermore, the model innovatively employed a random step sampling technique, which enabled the generation of 12,000 training samples using only 10% of the original dataset, substantially reducing dependence on complete aging data. From an industrial perspective, this approach demonstrated significant value: it could predict the full life cycle behavior using merely 20% of early-stage experimental data, thereby reducing experimental costs by 80%. This achievement established a dual-driven framework combining physical principles with data intelligence, offering a novel paradigm for polymer aging prediction.

Aref Ghaderi and colleagues [109] accurately predicted the degradation of mechanical properties in cross-linked elastomers during thermal oxidation and hydrolytic aging using the CondNN framework. This framework transformed the complex three-dimensional stress–strain tensor into multiple one-dimensional problems and employed 21 teams of intelligent agents to collaboratively model the evolution of the material’s microstructural network. By integrating physical constraints such as strain energy functions, microsphere-based dimensionality reduction, and network decomposition, the model ensured thermodynamic consistency and effectively captured stress softening in natural rubber, permanent deformation in polyurethane, and relaxation behavior in silicone. Experimental validation demonstrated that the model achieved a prediction error consistently below 10% for strength attenuation in SBR and degradation behavior in PLA-PCL under hydrolytic aging, with computational efficiency three times greater than that of conventional micromechanical models. This study provided a high-precision, data-efficient, and broadly applicable tool for polymer lifetime assessment across diverse application scenarios.

#### 3.4.4. Innovative Model Fusion Paradigm: Hierarchical Strategy for Polymer Adaptation Pathways

Hierarchical fusion improves the robustness of predictions by integrating multi-source data. In the future, within the field of polymer research, a joint input matrix can be developed that incorporates chemical structures (e.g., functional group types, molecular weight distributions), environmental parameters (e.g., temperature, humidity, ultraviolet irradiation), and microscopic morphological features (e.g., SEM/TEM images).

The future breakthrough resides in the advancement of cross-scale feature mapping algorithms, exemplified by the integration of graph attention networks to characterize the relationship between molecular chain mobility and macroscopic crack propagation. Concurrently, these algorithms can be coupled with synchrotron X-ray tomography to capture the evolution of nanoscale pores, thereby addressing the critical challenge of data registration. For instance, in future research, FTIR spectra and thermogravimetric data can be integrated through a graph neural network to elucidate the interplay between functional group degradation and thermal stability. However, the spatiotemporal misalignment of polymer data across molecular to macroscopic multiscale levels may undermine the effectiveness of early fusion. In contrast, while late fusion can integrate outputs from both mechanistic and data-driven models, it tends to overlook information on cross-modal interactions.

#### 3.4.5. Automation and Optimization Techniques: Adaptive Weights and Joint Parameter Tuning

In the process of improving model accuracy for predicting the aging life of polymers, particularly concerning the initiation and propagation of cracks, optimizing hyperparameters for complex neural network architectures—such as hybrid models combining CNN and LSTM—represents a key challenge [110,111]. Researchers are increasingly utilizing joint search strategies based on Bayesian optimization to address this challenge. The primary advantage of this approach resides in its global optimization capability and sampling efficiency. It constructs a probabilistic surrogate model of the objective function and utilizes this model to guide the selection of efficient sampling points. Notably, this strategy enables the “joint” and “synchronous” optimization of multiple types of key hyperparameters. This encompasses the convolutional kernel size, the number of layers, stride configuration, and selection of activation functions in the CNN, which partially influence the feature extraction capability; the number of hidden layer units, the number of layers, and the connection mode in the LSTM, which determine the effectiveness of temporal modeling; as well as the architectural parameters of the fusion module and global training parameters. Through intelligent collaborative search, Bayesian optimization enables systematic exploration of the parameter combination space, allowing for precise identification of the optimal configuration that maximizes the utilization of data features to predict crack evolution trends and identify potential failure risk points. This significantly enhances the overall accuracy and robustness of the hybrid model in predicting microscopic cracks in polymer materials, surpassing the limitations in efficiency and effectiveness of traditional grid or random search methods.

Adaptive weight design dynamically adjusts the contribution weights assigned to physical models and data-driven models through a gating network. For example, under high-temperature conditions, greater weight is given to the Arrhenius equation, whereas in high-humidity environments, the contribution of the data-driven model is increased. This method could substantially reduce the costs associated with manual trial and error and improve environmental adaptability; however, it requires a significant number of computational resources and exhibits reduced interpretability.

Finally, we condensed and summarized the methods mentioned in Chapter Three, as shown in Table 6:

While the cited case studies demonstrate the considerable potential of machine learning in predicting material aging, a critical analysis reveals that the transition from controlled laboratory settings to real-world operating conditions remains a primary challenge. The performance of data-driven models is intrinsically constrained by data limitations, including the scarcity of long-term aging data, heterogeneity across material scales, and biases inherent in accelerated testing protocols. For instance, models trained on accelerated aging data may fail to generalize under field conditions due to unaccounted-for stochastic stressors or multi-mechanism synergies. To enhance robustness, future efforts must prioritize the development of hybrid models that integrate physical laws, adopt dynamic data assimilation from in situ monitoring, and explore transfer learning across materials to mitigate data scarcity. Ultimately, the real-world efficacy of these models depends on embedding physical realism and overcoming the qualitative and quantitative limitations of current datasets.

## 4. Summary and Outlook

Machine learning has instigated a paradigm shift in polymer aging life prediction, overcoming the simplifications and constraints of traditional models to establish an intelligent framework integrating multi-physical field coupling and dynamic data. SVM models, grounded in structural risk minimization, excel in data-scarce scenarios. Neural networks enable cross-scale modeling from microstructural evolution to macroscopic failure through deep nonlinear transformations and multi-source data fusion. Decision tree models, leveraging interpretable feature partitioning and ensemble techniques, quantify the mechanisms of multi-stress field interactions.

Nevertheless, three fundamental challenges persist. Firstly, data-driven approaches inadequately integrate physical mechanisms, limiting their capacity to capture intrinsic feedback between material constitutive behavior and aging dynamics. Secondly, disrupted cross-scale correlations result in the absence of a comprehensive mathematical framework characterizing causal links from molecular motion to macroscopic failure. Thirdly, model generalizability remains constrained in extreme environments and novel material systems due to data distribution shifts and a lack of embedded physical constraints.

Future breakthroughs need to be centered around three dimensions (Table 7):

Through the above-mentioned integrated innovation, machine learning will drive the prediction of polymer lifespan to leap from empirical extrapolation to mechanism simulation, ultimately forming a new generation of intelligent prediction paradigms that are physically rigorous, dynamically adaptive, and universally applicable in engineering, laying a theoretical foundation for the full life cycle health management of major equipment.

## Figures and Tables

**Table 1:** Limitations of Conventional Polymer Lifespan Prediction Methods and Corresponding Advantages of Machine Learning Approaches.

The Core Challenge of Traditional Methods	The Corresponding Solutions Provided by Machine Learning
The assumption of a single variable of temperature dependence	Modeling multi-factor nonlinear relationships
Poor generalization ability of the models	Powerful generalization and transfer capability
Difficult to handle high-dimensional heterogeneous data	Multi-source heterogeneous data fusion analysis
High risk in extrapolation prediction	Robust extrapolation with embedded physical knowledge
Weak mechanism revelation capability	Feature importance analysis and interpretability

**Table 2:** Summary of Representative Applications and Performance of the SVM Model for Polymer Aging Life Prediction.

Research Case	Application	Performance Index
Liu et al., 2022 [44]	Fatigue life prediction of rubber vibration isolators	R^2^ = 0.9766, RMSE = ±1.3 (dispersion band), Accuracy = 92.5%
Zhang et al., 2015 [45]	Classification of SEM images for wheat straw/polypropylene composites	Classification accuracy = 92.5%, Sensitivity = 81.3–100% (for mid-term aging)
Mohanty et al., 2023 [46]	Monitoring the oxidative stability of BR	Low error in the Mooney viscosity model, accurate color index prediction

**Table 3:** Summary of Representative Applications and Performance of the ANN Models for Polymer Aging Life Prediction.

Research Case	Application	Performance Index
Liu et al., 2014 [59]	Aging life prediction of polycarbonate	Time error ≤ 15 days (within 7 months total aging time)
Doblies et al., 2019 [60]	Predicting thermal exposure behavior of epoxy resin	Residual strength prediction error = 1.35 MPa (NMAE = 4.51%)
Zhang et al., 2023 [64]	Predicting UV aging life of PMMA	High prediction accuracy (R^2^ > 0.99), low life prediction error

**Table 4:** Summary of Representative Applications and Performance of the CNN Models for Polymer Aging Life Prediction.

Research Case	Application	Performance Index
Li et al., 2025 [67]	Fatigue life prediction of rubber materials	Training accuracy = 99.08%, Test accuracy = 96.14%
Meng et al., 2024 [68]	Failure detection of epoxy coatings	Overall accuracy = 82.81%, High precision in crack identification
Zhang et al., 2019 [70]	Degradation detection of photovoltaic backsheets	Pixel accuracy = 92.8%, mean intersection over union = 72.5%

**Table 5:** Summary of Representative Applications and Performance of the Decision Tree-Based Models for Polymer Aging Life Prediction.

Research Case	Application	Performance Index
Liu et al., 2022 [88]	Contact fatigue life prediction of polymer gears	100% prediction accuracy (within a 3× dispersion band)
Nasiri et al., 2024 [86]	Fatigue life prediction of 3D-printed PLA biomaterials	R^2^ = 0.9766, Effective interpretation via SHAP analysis
Lu et al., 2025 [87]	Fatigue life prediction of polymer gears (hybrid data)	Error within 5× dispersion band, effective SHAP analysis

**Table 6:** Summary of Core Characteristics of Machine Learning Models for Polymer Aging Life Prediction.

Model Category	Typical Application Case	Model Advantages	Primary Limitations
Support Vector Machine	Rubber fatigue life prediction, aging stage classification of composites via SEM images, and antioxidant performance assessment.	Excellent small-sample adaptability, strong nonlinear mapping capability, ensures global optimum via structural risk minimization, robust to noisy data.	Performance is heavily dependent on feature engineering, sensitive to parameter tuning, and has poor model interpretability.
Neural Networks	Epoxy resin residual strength prediction, performance evolution of asphalt mixtures, coating crack identification, and multi-factor coupled rubber fatigue prediction.	Powerful nonlinear modeling capacity, autonomously learns features from multi-source heterogeneous data, supports dynamic data updating, and cross-scale modeling.	Requires large volumes of labeled data, high training cost; “black-box” limits mechanistic interpretability; generalization ability for new materials/environments needs improvement.
Decision Tree Models	Bending fatigue prediction of polymer films for flexible electronics, life assessment of 3D-printed PLA biomaterials, and contact fatigue prediction of polymer gears.	Intuitive model structure with strong interpretability, insensitive to missing values and outliers, effectively captures coupled effects of multi-stress fields.	Individual trees are prone to overfitting, poor handling of continuous variable gradients, and difficulty in automatically establishing cross-scale correlations from molecular to macroscopic properties.
Hybrid and Integrated Model	Probabilistic fatigue life prediction of composites, non-destructive assessment of coating porosity, prediction of thermo-oxidative/hydrolytic aging in elastomers.	Integrates physical mechanisms with data-driven approaches to enhance extrapolation reliability; GAN alleviates small-sample data bottlenecks; PINN embeds physical law constraints.	Complex model architecture with high computational overhead; deep integration of physical constraints with data-driven learning remains challenging; physical authenticity of generated data requires verification.

**Table 7:** Future Research Directions for Polymer Aging Life Prediction.

Research Level	Core Development Focus	Technical Approach	Expected Impact/Outcome
Theoretical	Develop a hybrid “physical information and data” intelligent framework.	Embed physical prior knowledge as regularization terms into the loss function of machine learning models.	Constrains the solution space of ML models, enhancing their physical consistency and reliability for extrapolation.
Methodological	Create cross-scale joint learning architectures.	Seamlessly integrate molecular dynamics simulations with continuum models using graph neural networks.	Establishes predictive pathways from molecular chain dynamics to macroscopic crack propagation, transforming prediction from empirical extrapolation to mechanism-based simulation.
Application	Build resource-efficient, adaptive prediction systems.	Combine transfer learning with online knowledge distillation techniques for deployment on edge computing devices.	Enables real-time modeling of complex environmental responses in the field, bridging the gap between lab models and industrial implementation for condition-based maintenance.

## Data Availability

No new data were created or analyzed in this study.
